# Liver Biopsy Hydroxyproline Content Is a Diagnostic for Hepatocellular Carcinoma in Murine Models of Nonalcoholic Steatohepatitis

**DOI:** 10.3390/diagnostics10100784

**Published:** 2020-10-04

**Authors:** Tyler L. Bissoondial, Yiguang Han, Stephanie Mullan, Amrit K. Pabla, Kiera Spahn, Steven Shi, Lana Zheng, Ping Zhou, Kai Jiang, Natalia Prakash, Shraddha Bhutkar, Quaisar Ali, Jingsong Li, Zhijian Hu, Anthony J. Pellicano, Itzhak D. Goldberg, Prakash Narayan

**Affiliations:** Department of Preclinical Research, Angion Biomedica Corp., 51 Charles Lindbergh Boulevard, Uniondale, NY 11553, USA; tylbissoondial@bmchsd.org (T.L.B.); infobox001@gmail.com (Y.H.); stephanieannmullan@gmail.com (S.M.); pablamrit@gmail.com (A.K.P.); kspahn5@gmail.com (K.S.); steven.0528.ss@gmail.com (S.S.); lana1zheng@gmail.com (L.Z.); pzhou@angion.com (P.Z.); kjiang@angion.com (K.J.); nprakash@angion.com (N.P.); shraddha.bhutkar@gmail.com (S.B.); quaisar.ali@gmail.com (Q.A.); jli@angion.com (J.L.); hzhijian@angion.com (Z.H.); apellicano@angion.com (A.J.P.); igoldberg@angion.com (I.D.G.)

**Keywords:** NASH, fatty, nonalcoholic, liver, HCC, cancer, hydroxyproline, ROC, diagnostic

## Abstract

There is increasing evidence that nonalcoholic steatohepatitis (NASH) is a risk factor for hepatocellular carcinoma (HCC) in the absence of cirrhosis, a phenomenon termed noncirrhotic HCC. Early diagnosis of HCC is critical to a favorable prognosis. We tested the hypothesis that hydroxyproline content of liver biopsy samples is diagnostic for HCC in murine models of NASH induced by diet or by diet and chemicals. The training set comprised mice fed a standard diet or a fast-food diet with or without administration of thioacetamide. At harvest, livers from the modified diet cohort exhibited NASH with a subset of NASH livers exhibiting HCC. Hydroxyproline content was measured in liver biopsy samples with tissue in the NASH+HCC cohort sampled from the remote, nontumor parenchyma. Plotting the receiver operating characteristics (ROC) with hydroxyproline as the continuous variable against the absence or presence of HCC yielded an area under ROC of 0.87, a threshold of >0.18 μg hydroxyproline/mg liver and sensitivity of 91% with a specificity of 83.3%. The use of liver hydroxyproline content as a diagnostic for HCC in a test set comprising healthy, NASH and NASH+HCC livers proved 87% accurate.

## 1. Introduction

Given the prevalence of diabetes, obesity and metabolic syndrome, the incidence and prevalence of nonalcoholic fatty liver disease (NAFLD) are increasing [1,2]. The NAFLD spectrum starts with simple steatosis or lipid deposition within the liver. Left untreated, steatosis can progress to nonalcoholic steatohepatitis (NASH), i.e., steatosis accompanied by inflammation of the liver, NASH with fibrosis, cirrhosis and finally hepatocellular carcinoma (HCC) [3,4,5,6,7,8]. Considering that ~30% of the population in the United States alone has some steatosis, the risk for a NASH epidemic looms large [1]. In fact, it is estimated that NAFLD-related hepatic complications will emerge as the leading cause of liver transplantation in the United States over the next 2 or 3 decades [1,2,3]. 

Historically, cirrhosis, regardless of etiology, carries the highest risk for HCC [9,10,11,12]. Emerging nonclinical and clinical evidence suggests that NASH can progress to HCC in the absence of cirrhosis, a phenomenon termed noncirrhotic HCC [3,4,5,6,7,8]. Given the sheer magnitude of the NAFLD epidemic, there is an increasing risk for NASH-related noncirrhotic HCC. Diagnosing HCC early is paramount as the outcome in this population remains grim with advancing disease [9,10,11]. While cirrhotics are routinely screened for HCC, the NASH patient is seldom screened for liver cancer. This is unfortunate considering that a) NASH is emerging as a cause of noncirrhotic HCC and b) NASH is a biopsy-proven label [13]. Liver biopsy tissue used for diagnosing NASH could also be purposed for diagnosing HCC. Although liver biopsy followed by microscopic evaluation remains the gold standard for diagnosing HCC [3,13], it is possible that the presence of remote tumor is missed during biopsy, resulting in potential misdiagnosis. 

The present study tested the hypothesis that liver biopsy hydroxyproline content is a diagnostic for HCC in NASH. Hydroxyproline, a major component of collagen, can be assayed biochemically, is not subject to observer bias and is routinely assayed to estimate liver fibrosis [3,14,15]. There is also evidence to suggest that excessive collagen deposition is associated with the transformation of the scarred liver an oncological phenotype [3,16,17]. Liver hydroxyproline content was evaluated in murine models of NASH with or without HCC. Disease was induced in mice with the administration of a modified diet or the modified diet with chemicals, both of which have been reported to demonstrate the entire spectrum of NAFLD culminating in HCC [3,15,17,18,19,20].

## 2. Materials and Methods

### 2.1. Animal Models

All in-life studies were conducted in adult male or female C57BL/6 mice (18–20 g, ∼6 weeks old) after approval (5 May 2019, #2019-014) from our Institutional Animal Care and Use Committee (IACUC). Food and drink were provided ad libitum.

The training set (*n* = 39) comprised mice randomized to 3 groups [3,15,18,19,20]. A sham cohort comprised animals on a standard rodent diet for either 5 mo (*n* = 4) or 17 mo (*n* = 9). A fast-food diet (FFD) cohort comprised mice (*n* = 15) on a modified rodent diet containing 40 kcal % fat, 20 kcal % fructose and 2% cholesterol (D09100301, Research Diets, New Brunswick, NJ, USA) for 17 mo. An FFD + thioacetamide (TAA) cohort (n = 11) was provided FFD for 5 mo and injected with TAA (100 mg/kg, intraperitoneal (IP) × 3/week) over the first 2 mo. The test set (*n* = 15) comprised animals randomized to a standard diet for up to 17 mo (*n* = 6), an FFD diet (*n* = 2, 17 mo) or an FFD diet + TAA (*n* = 4, FFD 5 mo, TAA, 2 mo) or FFD diet + CCl_4_ + (0.32 μg/g, IP × 1/week) and 18.9 g/L d-glucose (drinking water) for 3 mo (*n* = 3). At sacrifice, animals were anesthetized with ketamine/xylazine (25/5 mg/kg, IP), blood was withdrawn, and livers were harvested. 

### 2.2. Histopathology

Histopathological analysis was conducted by several observers blinded to the identity of the groups. At sacrifice, a portion of the liver was fixed in formalin (10%) and stained with hematoxylin & eosin (H&E), evaluated for steatosis, inflammation and ballooning, and the NAFLD activity score (NAS) computed [3,19]. This scoring system on the 0–8 scale (8 being most diseased) totals the individual component scores for steatosis (0–3), lobular inflammation (0–3) and hepatocyte ballooning (0–3). For each liver, the NAS was averaged across several observers. Picrosirius red (PSR)-stained liver sections were semiquantified by blinded observers and averaged for each liver to estimate extracellular fibrillar collagen using ImageJ (https://imagej.nih.gov/ij/). Several fields per liver were evaluated to ensure that data were representative of that liver.

At sacrifice, livers were examined by two or more independent observers for the absence or presence of tumors. Diagnosis of HCC was made under microscopic observation in H&E-stained liver sections by trained and independent observers as previously reported [3,16]. Healthy liver parenchyma or parenchyma interrupted by steatosis, inflammation and ballooning but lacking HCC features were classified as HCC-free [3]. To confirm diagnosis of HCC, liver sections were stained with cytokeratin 7 staining (anti-Cytokeratin 7 antibody (RCK105), Abcam, San Francisco, CA, USA), which localizes to neoplasms [16], and carbohydrate antigen (CA) 19-9 staining (anti-CA-19-9 antibody, orb27274, Biocompare, South San Francisco, CA, USA) as previously reported [16].

### 2.3. Liver Function Tests

Serum samples were sent to the Northwell Health (Lake Success, NY, USA) for determination of aspartate aminotransferase (AST) and alanine aminotransferase (ALT).

### 2.4. Liver Hydroxyproline

At sacrifice, hepatic hydroxyproline content was evaluated [14] in remote, nontumor liver sections snap-frozen in liquid N_2_ and stored at −80 °C until analysis. Tissue was weighed and then homogenized in 500 μL water. Five hundred microliters of HCl (10.0 N) was added to the samples and hydrolyzed at 120 °C for 3 h. Supernatants were transferred to a 96-well plate, and wells were allowed to evaporate dry. Hydroxyproline content was determined by colorimetric (catalog # MAK008, Sigma Aldrich, St. Louis, MO, USA) analysis and expressed as µg hydroxyproline/mg liver.

### 2.5. Data Analysis

Data are presented as mean ± standard error or mean. One-way analysis of variance followed by Tukey’s post-hoc test was used to compare data between groups. A *p* < 0.05 was deemed significant. Receiver operating characteristic (ROC) curves, i.e., true positive rate (sensitivity) vs. false positive rate (1-specificity), were generated in Excel using Pivot Tables and area under ROC (AUROC) calculated using the trapezoidal method. The threshold was selected, and sensitivity (S_n_) and specificity (S_p_) at this threshold were identified.

## 3. Results

**Figure 1:**
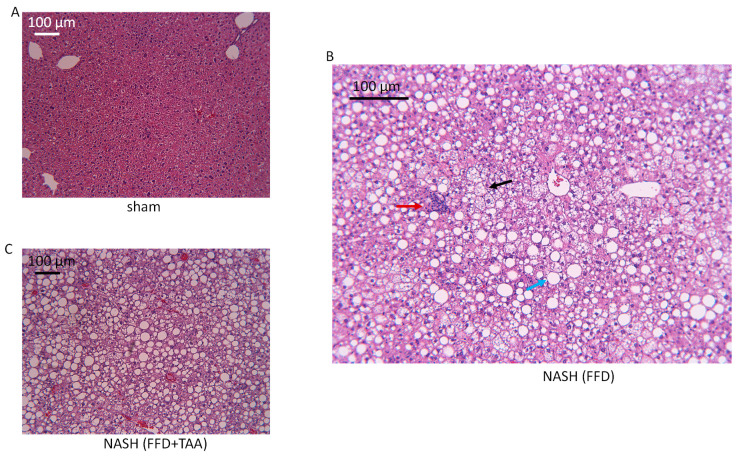
Nonalcoholic Fatty Liver Disease (NAFLD). Representative images (10× of H&E-stained liver sections from mice randomized to a standard diet (**A**, sham), fast-food diet (FFD) (**B**) or FFD+TAA (**C**). The blue arrow (**B**) shows one of many lipid droplets, the red arrow shows a site of inflammation and the black arrow shows hepatocyte ballooning.

Within the training set, H&E stained liver sections from the FFD and FFD+TAA cohorts revealed hallmark characteristics of NASH, including steatosis, inflammation and ballooning (Figure 1). Indeed, steatosis was the prominent feature in both the FFD and FFD+TAA cohorts.

**Figure 2:**
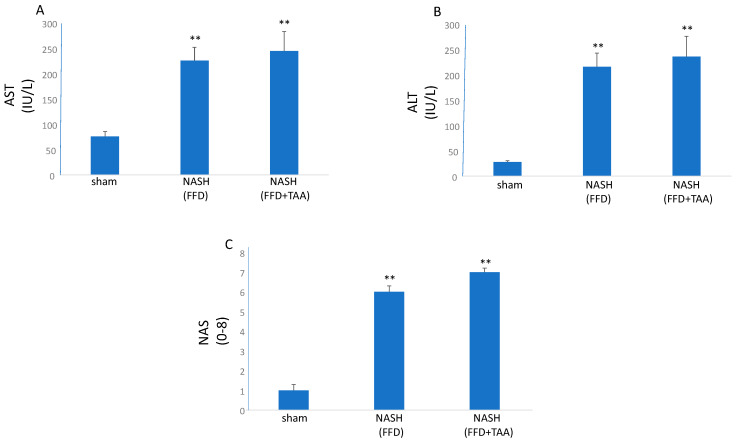
Nonalcoholic steatohepatitis (NASH). (**A**) and (**B**), Liver function tests and (**C**) NAFLD Activity Score (NAS) were elevated in the FFD and FFD+TAA cohorts compared to sham. ** *p* < 0.01 vs. sham.

Consistent with NASH, both AST and ALT were elevated multifold in both the FFD and FFD+TAA groups (Figure 2). Evaluation of NAS in H&E-stained liver sections demonstrated an increase in this score compared to the sham cohort (Figure 2).

**Figure 3:**
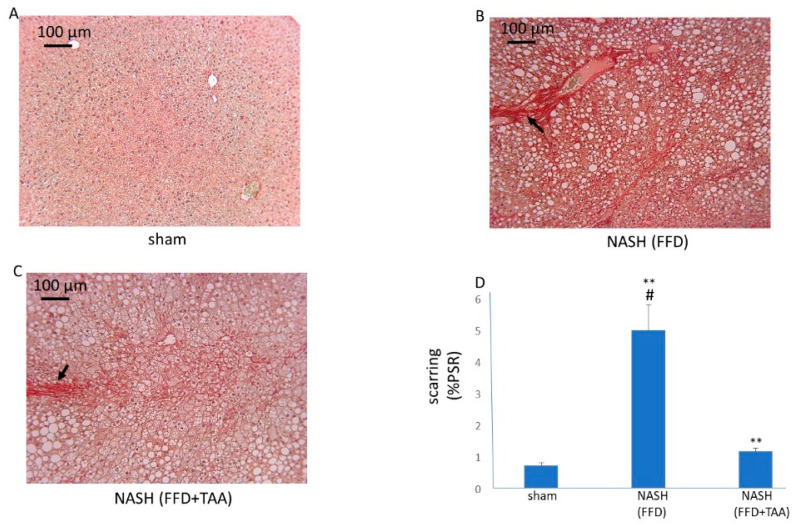
Liver scarring. Representative images (10×) of PSR-stained liver sections from mice randomized to a standard diet (**A**), FFD (B) or FFD+TAA. (**C**) The black arrow (**B**) shows bridging fibrosis. (**D**) Quantitation of PSR staining shows elevated scarring in the FFD and FFD+TAA cohorts. ** *p* < 0.01 vs. sham. # *p* < 0.01 vs. FFD+TAA.

To determine whether scarring was present in livers from the modified diet ± TAA groups, PSR staining, which enables visualization and estimation of collagen, was used. A filigree network of PSR staining was evident in livers from both the FFD and FFD+TAA cohorts with livers from FFD (17 mo) cohort exhibiting the brightest staining and therefore the highest amount of collagen deposition (Figure 3).

**Figure 4:**
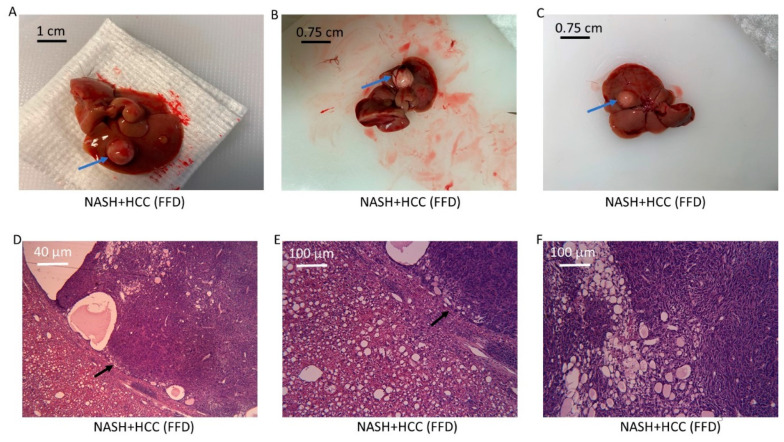
NASH with hepatocellular carcinoma (HCC). (**A**–**C**) Several animals within the FFD (17 mo) cohort exhibited liver tumors, which manifested as one or more polyp-like (blue arrows) growths on the liver. (**D**–**F**) Representative H&E-stained section from an FFD (17 mo) liver showing a trabecular growth pattern of atypical hepatocytes and clusters of nuclei (F, 25×) with a distinct margin between the noncancerous and cancerous parenchyma (black arrows, (D) (4×) and (E) (10×)). This liver bore a large tumor at sacrifice. Steatosis is also evident (**E**).

Within the training set, at sacrifice, several livers from the FFD (17 mo) cohort exhibited one or more large tumors (Figure 4), a finding consistent with a previous report from this laboratory. No tumors were evident in either the sham or the FFD+TAA (5 mo) cohorts (data not shown). To confirm that these tumors were indeed HCC, microscopic examination of liver sections housing the tumors was conducted. Once again, consistent with a previous report from this laboratory, livers that presented with tumors on gross observation presented with a trabecular growth pattern of atypical hepatocytes and clusters of multinucleated hepatocytes evident in H&E-stained sections (Figure 4). In fact, a distinct margin was observed between the cancerous and noncancerous parenchyma.

**Figure 5:**
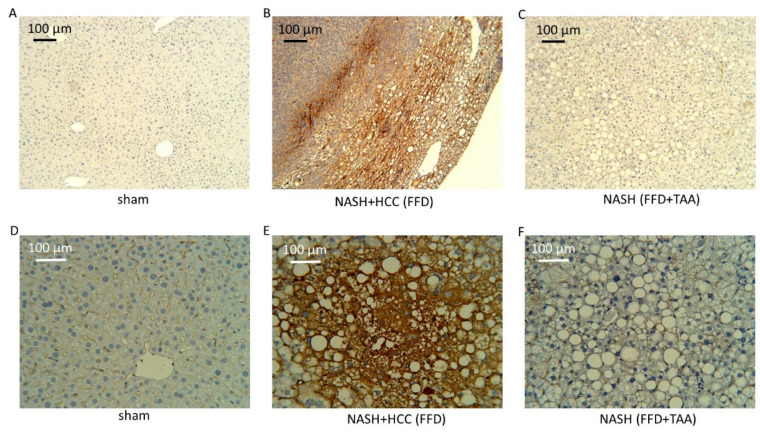
HCC. (**A**–**C**) Representative liver sections (10×) from Ca-19-9-stained sham, FFD and FFD+TAA groups. Staining is enriched in the FFD liver indicative of HCC. This liver bore a large tumor at sacrifice. (**D**–**F**) Representative liver sections (25×) from cytokeratin-7-stained sham, FFD and FFD+TAA groups. Staining is enriched in the FFD liver, indicative of HCC. This liver bore several tumors at sacrifice.

Additionally, liver sections housing tumors were stained with CA 19-9 or cytokeratin 7 or markers of gastrointestinal carcinoma. Indeed, as reported previously, enriched CA 19-9 and cytokeratin 7 staining (Figure 5) was observed in sections from livers bearing tumors. By contrast, livers that were tumor-free, be it from the sham cohort or the NASH cohort, exhibited little or no CA 19-9 or cytokeratin 7 staining.

**Figure 6:**
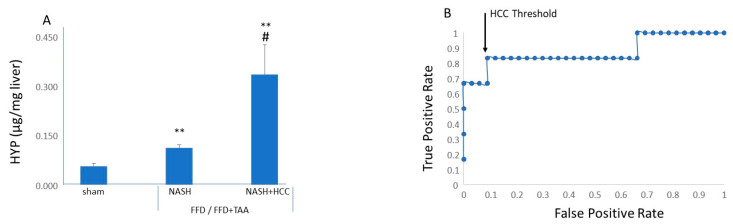
Liver hydroxyproline as a diagnostic for HCC. (**A**) Compared to the sham cohort, biopsies from livers characterized as NASH (FFD or FFD+TAA) had increased hydroxyproline content. ** *p* < 0.01 vs. sham. Hydroxyproline content was highest in biopsies from NASH+HCC livers. # *p* < 0.01 vs. NASH. (**B**) Receiver operating characteristics for liver hydroxyproline as a diagnostic for HCC in NASH. The threshold was selected as further improvement in sensitivity is associated with increased false positives.

Hydroxyproline content was measured in remote (nontumor) biopsies in all three cohorts. Compared to the sham cohort, biopsies from NASH livers (FFD or FFD+TAA) exhibited increased hydroxyproline (Figure 6). However, tissue from livers bearing tumors, i.e., the NASH+HCC livers, had the highest hydroxyproline content. Plotting the ROC curve with hydroxyproline as the continuous variable against the absence or presence HCC (binary outcome) yielded an auroc of 0.87. A threshold of >0.18 μg hydroxyproline/mg liver was selected as it yielded the highest sensitivity accompanied by the lowest false positive rate. For this threshold, sensitivity was 91% with a specificity of 83.3%. 

Livers from the test set of animals were assessed for the absence or presence of HCC as described above (data not shown). Of the 15 animals comprising the test set, nine animals were HCC-negative and six were HCC-positive. Hydroxyproline content was measured in liver biopsy samples from the test set. The use of liver biopsy hydroxyproline content as a diagnostic for HCC yielded 13/15 or 86.6% animals as being correctly diagnosed as HCC-negative or -positive (Table 1). Two animals that did not have HCC were classified as HCC-positive by the diagnostic (false positive).

## 4. Discussion

In mice fed an FFD with or without chemical injury, NASH with scarring was evident, with some animals developing HCC. Liver biopsy hydroxyproline content was elevated in NASH with further elevation in the NASH+HCC cohort. The use of a supervised learning approach in our models of NASH indicates that a liver biopsy hydroxyproline content >0.18 μg/mg tissue is an excellent diagnostic for HCC in NASH with an AUROC of 0.87, s sensitivity of 91% and a specificity of 83.3%. These data suggest that hydroxyproline determination in a remote, nontumor NASH liver biopsy sample may be used to diagnose HCC.

When diagnosed late, HCC is almost always uniformly fatal [11]. The highly vascularized hepatic bed facilitates tumor growth while making tumor resection extremely challenging. In fact, Barcelona Clinic Liver Cancer (BCLC) stages B-D are deemed noncurative [11]. Historically, cirrhotics are at the highest risk for HCC, with hepatitis B or C and/or alcoholism being the leading causes of cirrhosis [3]. Consequently, cirrhotics are regular screened for HCC; ultrasonography with or without serum alpha-fetoprotein level is used to diagnose HCC in this population [3,9,10,11,12]. Emerging nonclinical and clinical reports indicate that patients presenting with NASH, especially those presenting with NASH with fibrosis, are also at increased risk of HCC [3,4,5,6,7,8]. In fact, obesity, metabolic syndrome and type 2 diabetes, are each related, and pivotal factors in NAFLD. The most important consequence of these epidemics is the probable rise in the incidence of HCC [21]. The clinical findings are especially alarming as a subset of this population presents with HCC absent cirrhosis, a phenomenon termed noncirrhotic NASH. Second, given the sheer size of the NAFLD epidemic—30% of the United States population with some steatosis, ~10–12 million of those with NASH and 2–5 million of those with fibrosis [1,2]—even a small percent incidence of HCC translates to staggering numbers. Unlike cirrhotics, NASH patients are rarely screened for HCC. This is especially unfortunate given that NASH itself is a biopsy-proven label, and typically two or three tissue samples are obtained during the biopsy procedure [13]. Microscopic examination of liver tissue remains the gold standard for diagnosis of HCC in NASH as ultrasonography, especially in obese patients, may miss early, smaller tumors. Nonetheless, given the attendant hepatomegaly in the fatty liver, there is a distinct possibility that needle biopsy might also miss lurking tumors. 

The present study was conducted in murine models of NASH queried livers on the NAFLD continuum for the absence or presence of HCC as a function of liver biopsy hydroxyproline content. The supervised learning approach employed a training set [3,15,18,19] comprising mice on a standard diet or an FFD diet ± TAA for varying time intervals. As reported previously, C57BL/6 mice administered FFD alone exhibit the entire continuum of NAFLD, culminating in HCC [3]. In addition to FFD, the use of chemicals such as TAA induces and accelerates this program in part by increasing oxidative stress and upregulation of lipid peroxidation [22,23,24]. Findings from this study are consistent with other reports indicating that livers from animals randomized to this regimen exhibited hallmark signs of NASH with scarring evidenced under microscopy (H&E and PSR staining) and by increased liver function test values [3,15,19,20]. Also consistent with a previous report from this laboratory [3], a subset of animals in the 17 mo FFD cohort exhibited hepatic tumors subsequently confirmed by H&E microscopy and cytokeratin 7 and CA 9-19 immunohistochemistry [16]. Interestingly, compared to the sham cohort liver biopsy, hydroxyproline content was increased in the NASH cohort, with animals exhibiting NASH+HCC demonstrating the highest hepatic hydroxyproline content. This is not entirely unexpected in that hydroxyproline is a constituent of collagen, and previous reports [3,16] from this laboratory point to the transformation of the scarred liver to an oncotic phenotype. Indeed, a preponderance of literature [9,10] suggests that cirrhosis is the highest risk factor for HCC, albeit livers in this study presented with noncirrhotic HCC. Analysis of the ROC curve suggests that a biopsy with >0.18 μg hydroxyproline/mg liver has an ~87% probability of originating from an HCC liver. Evaluation of liver hydroxyproline as a diagnostic for HCC was conducted in a test set comprising animals with healthy livers, NASH livers and NASH+HCC livers. Liver pathology within the test cohort was induced with FFD, FFD+TAA or FFD+CCL_4_+glucose, the last regimen associated with necrosis of centrilobular hepatocytes, the activation of Kupffer cells and the induction of an inflammatory response [21,22,23]. The use of hydroxyproline as a diagnostic for HCC was associated with 87% accuracy, with only 2 out of 15 liver returning false positive values.

These empirical findings may be clinically relevant in that they can eventually lead to a quantitative framework for making a diagnosis of HCC in liver biopsy samples from NASH patients. Liver hydroxyproline content is routinely measured as a marker of scarring [3,14,15]. Importantly, in this study, hydroxyproline was sampled in biopsies from remote, nontumor tissue; the tumor bed did not require sampling, therefore adding translational significance to these findings. The hepatic hydroxyproline threshold for HCC is expressed as a function of sampled tissue mass, making the entire exercise self-contained and independent of the mass of the entire liver. Hydroxyproline is extremely stable and can be quantified equally well from frozen samples or formalin-fixed samples [14]. Hydroxyproline content in a liver biopsy sample is a laboratory number that is not subjected to observer bias. Finally, while not necessarily point-of-care, the hydroxyproline proline assay [14] is less labor-intensive than the preparation of and reading of slides for microscopic determination of NASH and/or HCC. 

This study does have weaknesses in that findings are made from and therefore potentially restricted to a subset of murine NASH models. Needless to say, these findings need to be validated in additional models of liver disease. For these findings to be clinically applicable, prospective trials in NASH and NASH-related HCC patients would need to be conducted and a ROC curve and threshold established in patient populations. Furthermore, while the concept of using liver biopsy hydroxyproline content as a diagnostic for HCC may hold, the threshold may very well be different in humans. Nevertheless, to the best of our knowledge, this is the first report demonstrating the utility of liver tissue hydroxyproline content as a diagnostic for HCC in NASH.

## Figures and Tables

**Table 1:** Evaluation of liver biopsy hydroxyproline as a diagnostic for HCC in NASH. Of the 15 livers comprising the test set, 9 were free of HCC and 6 were HCC-positive. Use of liver biopsy hydroxyproline as a diagnostic yielded and accuracy of 87%. Two livers were deemed HCC-positive when in fact they were not.

Sample	Tumor	HYP (μg/mg Liver)	HCC	False Positive
1	no	0.17	no	
2	no	0.16	no	
3	no	0.18	no	
4	yes	0.45	yes	
5	yes	0.50	yes	
6	no	0.55	yes	yes
7	yes	0.53	yes	
8	yes	0.40	yes	
9	yes	0.19	yes	
10	no	0.18	no	
11	no	0.23	yes	yes
12	no	0.16	no	
13	yes	0.30	yes	
14	no	0.18	no	
15	no	0.18	no	
